# A Molecular Biological and Biochemical Investigation on *Mycobacterium tuberculosis* MutT Protein

**DOI:** 10.5812/jjm.9367

**Published:** 2014-03-01

**Authors:** Hsiu-Lin Huang, Ho-Ting Su, Chung-Hsiun Herbert Wu, Jyy-Jih Tsai-Wu

**Affiliations:** 1 Department of Biotechnology, Ming Dao University, Chang Hua, Taiwan; 2 Agriculture and Food Agency, Council of Agriculture, Taipei, Taiwan; 3 Protein Engineering and Biologics Development I, Institute of Biologics, New Taipei, Taiwan; 4 Department of Medical Research, National Taiwan University Hospital, Taipei, Taiwan

**Keywords:** *Mycobacterium tuberculosis*, Protein Array Analysis, *Escherichia coli*

## Abstract

**Background:**

*Mycobacterium tuberculosis* is a vicious microbe co-existing with the infected host. This pathogen exploited opportunities to spread during periods of urbanization and social upheaval, and got retreated with improved hygiene.

**Objectives:**

This investigation was designed to clone and characterize *M. tuberculosis* *mutT* gene, a homologue of a DNA repair protein in *Escherichia coli*. The aim was to depict the possible role of this homologue in the virulent microbe.

**Materials and Methods:**

A DNA fragment of the *mutT* gene was amplified with PCR from the genomic DNA of strain H37Rv* M. tuberculosis*. The expression vector was transformed into *E. coli* strains BL21 (DE3) and MK602 (DE3) (*mutT*-). The protein activity assay was performed by biochemical methods.

**Results:**

*M. tuberculosis* MutT shares 23% identity with the *E. coli* MutT protein. The *mutT* gene DNA fragment was subcloned into the expression vector pET28a(+) and the recombinant plasmid was overexpressed in *E. coli*. Purified and refolded *M. tuberculosis* MutT possesses a dGTPase activity, which is one of the most well-known preference nucleotidase activities of MutT in *E. coli*. This study also showed that the dGTPase activity of *M. tuberculosis* MutT was enhanced by magnesium and inhibited by Ni^2+^ or EDTA. Endogenous MutT protein in *M. tuberculosis* lysate displayed a smear pattern in the Western blot, suggesting instability of this protein in the bacteria similar to the important proteins, such as P53 protein, tightly regulated by protein degradation.

**Conclusions:**

The cloned *M. tuberculosis* *mutT* gene and MutT protein were characterized. *M. tuberculosis* MutT has a dGTPase activity, which is one of the most well-known preference nucleotidase activities of MutT in *E. coli*. These findings provide further understanding about the vicious bacterium.

## 1. Background

Overexpression, purification and characterization of *Escherichia coli* *mutT* gene product indicated that MutT cannot bind to dsDNA or ss DNA and does not have any exonuclease activity (1, 2). Bhatnagar and co-workers (3), using different kinds of canonical nucleotides and several modified nucleotides as substrates, discovered that MutT has nucleoside triphosphatase activity on all canonical nucleoside triphosphates with a preference for dGTP. It was also reported (4-7) that MutT protein can hydrolyze 8-oxo-dGTP, an oxidatively damaged nucleotide and a potent mutagenic substrate for DNA synthesis. Such a protein might be particularly important for organisms living under excessively oxidative conditions.

*Mycobacterium tuberculosis* is an infectious microbe that co-exists with the infected host. It suffers severe oxidative stress produced by the host macrophage. As immunity wanes, through aging or immune suppression, the dormant bacteria can reactivate. Tuberculosis is the world’s leading cause of death from a single infectious agent, killing about 3 million individuals every year. About 1.7 billion people, roughly one-third of the world’s population, are infected with the causative agent *M. tuberculosis* (8). This pathogen has exploited opportunities to spread during periods of urbanization and social upheaval, and got retreated with improved hygiene. It has also weathered the antibiotic revolution and developed drug-resistant forms. Moreover, it has a lethal relationship with human immunodeficiency virus (HIV); during the early stages of HIV infection, susceptibility to tuberculosis increases, and tuberculosis in turn accelerates the progression to acquired immunodeficiency syndrome (AIDS). The complete genome sequence of a *M. tuberculosis* laboratory strain, H37Rv, was determined which has retained full virulence in animal models of tuberculosis and is also susceptible to drugs and amenable to genetic manipulation (9). Studies of H37Rv may improve our understanding of this slow-growing pathogen biology and help develop concepts of new prophylactic and therapeutic interventions.

In this study, we reported about cloning and characterization of *mutT* homologue in *M. tuberculosis*. The gene was overexpressed as a His-tagged recombinant form in *E. coli* *mutT*-mutant. The purified and refolded protein displayed dGTPase activity, which could be enhanced by an increasing concentration of magnesium. Ni^2+^ or EDTA both could inhibit dGMP hydrolysis. These findings may provide evidence for the correlation between the pathogen virulence and the DNA repair system.

## 2. Objectives

In this study, the activity of MutT protein in the virulent bacterium, *M. tuberculosis*, was investigated. The aim was to shed light on the possible role of the protein.

## 3. Materials and Methods

### 3.1. Bacterial Strains, Plasmids, and Antibodies

*E. coli* isogenic strain MK602 (leu^+^ *mutT*) was provided by Dr. M. Sekiguchi (2), and BL21 (DE3) was purchased from Novagen (Madison, WI). Plasmids pCR®II and pET28a (+) were obtained from Invitrogen (Carlsbad, CA) and Novagen (Madison, WI), respectively. Mouse monoclonal anti-histidine antibody was from Clontech (Palo Alto, CA) and horseradish peroxidase (HRP)-conjugated goat anti-mouse IgG antibody was from Santa Cruz Biotechnology (Santa Cruz, CA). HRP-conjugated donkey anti-rabbit IgG antibody, ECL kit, and Hyperfilm-MP film were obtained from Amersham Pharmacia Biotech (Buckinghamshire, England).

### 3.2. Cloning of *M. tuberculosis mutT* Gene

Most of the cloning procedures were performed following standard protocols (10). According to the published genomic sequence of *M. tuberculosis* (accession no. Z95584, AL 123456 CDS 24794-25219), two PCR primers were synthesized, HW 117 (5'-CATATGCTGAATCAGATCGTGGTTGCC-3') and HW 118 (5'-GGATCCTAACAGCGACGGTGGACATCT-3'). A DNA fragment containing the mutT gene was amplified with PCR from genomic DNA of *M. tuberculosis* strain H37Rv. PCR reaction (50.25 µL) contained 0.13 µg of template DNA, 0.1 µg of each primer, 0.2 mM dNTP, 4 mM Mg^2+^, and 3.75 units of Klen Taq DNA polymerase (Ab Peptides, St. Louis, MO). 

The PCR reaction was performed as follows: 95˚C for 2 minutes for denaturation; 30 cycles at 94˚C for 30 seconds, 60˚C for 30 seconds and 72˚C for 45 seconds for amplification; and a final incubation at 72˚C for 10 minutes for extension. The amplified products were analyzed in a 2% agarose gel. The PCR product was cloned into the pCR®II T-vector (Invitrogen). Both strands of the cloned fragment were sequenced with BigDye Terminator Ready Reaction Kit (Perkin Elmer, Alameda, CA) using ABI PRISM 310 Genetic Analyzer automatic sequencer (Perkin Elmer Applied Biosystems). *M. tuberculosis* *mutT* was subcloned into the Nde I and Bam HI sites of the expression vector pET28a (+) (Novagen) for overexpression of a histidine-tagged recombinant protein.

### 3.3. Lysogenization of MK602 (*mutT-*)

Integration of λDE3 prophage into the *E. coli* MK602 chromosome was performed with the λDE3 Lysogenization kit (Novagen, Madison, WI) according to the manufacture’s protocol. MK602 was grown in LB broth at 37˚C until OD600 = 0.5. Five mL of host cells were mixed with 108 pfu of λDE3, 108 pfu of helper phage, and 108 pfu of selection phage. For adsorption of the phage in to the host, the host/phage mixture was incubated at 37˚C for 20 minutes. The mixture was then spread onto an LB plate and incubated at 37˚C overnight. Bacterial candidates of λDE3 lysogen were picked from the plate and tested for suppression and induction of the T7 RNA polymerase gene. Two hundred mL of the candidate bacteria and 10 µL of the 10-8 fold diluted tester phage were mixed and incubated at 37˚C for 20 minutes, poured onto LB plates (with or without IPTG, MD Bio, Taiwan) with 2.5 mL molten top agar, and incubated at 37˚C overnight. The Lysogen, which gave the largest distinction of plaque sizes between tests with or without IPTG incubation, was chosen for the following experiments.

### 3.4. Overexpression of the Recombinant Protein

Overnight culture of BL21 (DE3) or MK602 (DE3) (*mutT*) bacteria harboring plasmid with *mutT* gene was inoculated into fresh medium and grown to an OD600 of 0.5-0.7 in LB broth containing 50 µg/mL kanamycin (MD Bio, Taiwan) at 37˚C. To induce the expression of the recombinant protein, IPTG was added to a final concentration of 1 mM and the bacteria were grown for an additional 4 hours at 37˚C (or 25˚C for MK602 [DE3]). The bacteria were harvested with centrifugation of 10,000 rpm for 10 minutes at 4˚C (JLA 10.5 rotor, Beckman Avanti centrifuge J-25).

### 3.5. Purification of the Recombinant *M. tuberculosis* MutT Protein

Complete disruption of bacteria was confirmed by direct microscopic examination. The bacterial lysate was then gradually brought to 20˚C in a 37˚C water bath to inactivate the activity of lysozyme. The protein fractions were saved at -70˚C for further purification. The histidine-tagged recombinant *M. tuberculosis* MutT protein was affinity purified with Ni^2+^-charged column (5 mL, Pharmacia Biotech, Sweden) in a FPLC system (Pharmacia Biotech LCC-500). The histidine-tagged protein was eluted from the column with 260-400 mM imidazole linear gradient. Purity of the fraction was examined by SDS-PAGE and the protein concentration was determined with the reagent of Bio-Rad Protein Assay (Bio-Rad, Hercules, CA) based on the protocol described by Bradford (11).

### 3.6. Antibody Preparation

Antiserum preparation was based on standard procedures with minor modifications (12). The recombinant *M. tuberculosis* MutT protein was fractionated in a SDS-polyacrylamide gel, then placed in a clean tray and negatively stained with ice-cold 0.25 M KCl. The gel piece containing the *M. tuberculosis* MutT protein was cut out and ground to pieces, each containing approximately 1 mg protein, were then mixed with Freund’s Complete Adjuvant (Imject®, Pierce, Rockfore, IL) and 1 × PBS at the ratio required to immunize a New Zealand white rabbit. Two boosts with protein gels containing 350 and 150 µg protein (mixed with Freund’s complete and incomplete adjuvant ]pierce[, respectively) were given at one-month intervals. Blood samples were collected one week after each boost. 

The collected blood was incubated at 37˚C for one hour, and the clog was teased off the tube wall using a spatula. After an overnight incubation at 4˚C, the clog was removed by centrifugation at 11,000 rpm for 10 minutes at 4˚C (RA-200J rotor, Kubota, Japan) and the supernatant was saved as the antiserum, which was aliquoted into small volumes and stored at -20˚C for long term storage. For routine use, the antiserum was stored at 4˚C in the presence of 0.02% sodium azide.

### 3.7. Western Blotting

Western Blotting was performed according to the standard protocol (12). Bacterial cell lysates or purified proteins were subjected to SDS-polyacrylamide gel electrophoresis, electrotransferred to nitrocellulose membrane (Optitran BA-SB3, Schleicher & Schuell, Germany) or polyvinylidene fluoride (PVDF) microporous membrane (Immobilon-P, Millipore, Bedford, MA), and detected with mouse or rabbit antisera, followed by 1:5,000 diluted horseradish peroxidase (HRP)-conjugated second antibody. The results were then detected with ECL kit (Amersham Pharmacia Biotech) and recorded on a Hyperfilm-MP film (Amersham Pharmacia Biotech).

### 3.8. In vitro Activity Assay

The dGTPase activity of the *M. tuberculosis* MutT was determined according to the procedure described by Akiyama (13). The *M. tuberculosis* MutT protein from MK602/pET28a (+)-*mutT* was incubated with 100 µM [α-32P] dGTP (2 Ci/mmol) (NEN, Boston, MA) in a buffer containing 20 mM Tris-HCl, pH = 7.5, 80 µg/ml BSA, 8 mM MgCl_2_, 5 mM DTT, and 4% glycerol at 30˚C for 20 minutes, and the reaction was stopped by addition of EDTA to the final concentration of 45 mM. An aliquot of the reaction mixture and unlabeled nucleotide standards were spotted on a PEI-cellulose sheet (Merck, Germany) and developed with 1 M LiCl in a closed chamber. The enzymatic activity was recorded with autoradiography and PhosphorImager (Fuji BAS-III, Japan) and quantified by the IMAGEGAUGE program (Fuji, Japan). The unlabeled standards were visualized by UV.

### 3.9. Denaturation and Renaturation of MutT Protein

Denaturation and renaturation (refolding) of the purified *M. tuberculosis* MutT protein were performed according to Hager and Burgess (14) and Tsai-Wu et al. (15) with some modifications. Recombinant MutT protein was Ni^2+^-charged-column purified, fractionated in a SDS-PAGE gel, and negatively stained with ice-cold KCl. The MutT protein (150 µg) containing gel was cut out and ground to pieces. The MutT protein was eluted from the gel pieces by immersing in 4 mL elution buffer (50 mM *Tris-HCl* (pH = 7.8), 0.1% SDS, 0.15 M NaCl, 0.1 mg/mL BSA), and rotated at 4˚C overnight. The eluted *M. tuberculosis* MutT protein in the supernatant was recovered by a centrifugation of 11,000 rpm for 1 minute at 4˚C (RA-200J rotor, Kubota, Japan). Four volumes of ice-cold acetone were added to the supernatant to precipitate the *M. tuberculosis* MutT protein. This mixture was inverted 6 times, and allowed to precipitate at -70˚C for one hour. The precipitated proteins were collected by centrifugation in RA-200J rotor (Kubota, Japan) at 11,000 rpm for 10 minutes, and the pellet was allowed to dry by putting the tube in an inverted position. The precipitated protein was dissolved in 400 µL of denaturation buffer (50 mM Tris-HCl [pH = 7.5], 0.1 mM EDTA, 1 mM DTT, 6 M guanidine-HCl, 150 mM NaCl, 20% glycerol and 0.1 mg/mL BSA). Renaturation of the *M. tuberculosis* MutT protein was performed by 3 consecutive dialysis against 80 mL of dilution buffer (50 mM *Tris-HCl* [pH = 7.5], 150 mM NaCl, 20% glycerol, 0.1 mg/mL BSA, 1 mM DTT and 0.1 mM EDTA) for at least two hours each time.

## 4. Results

**Figure 1.**
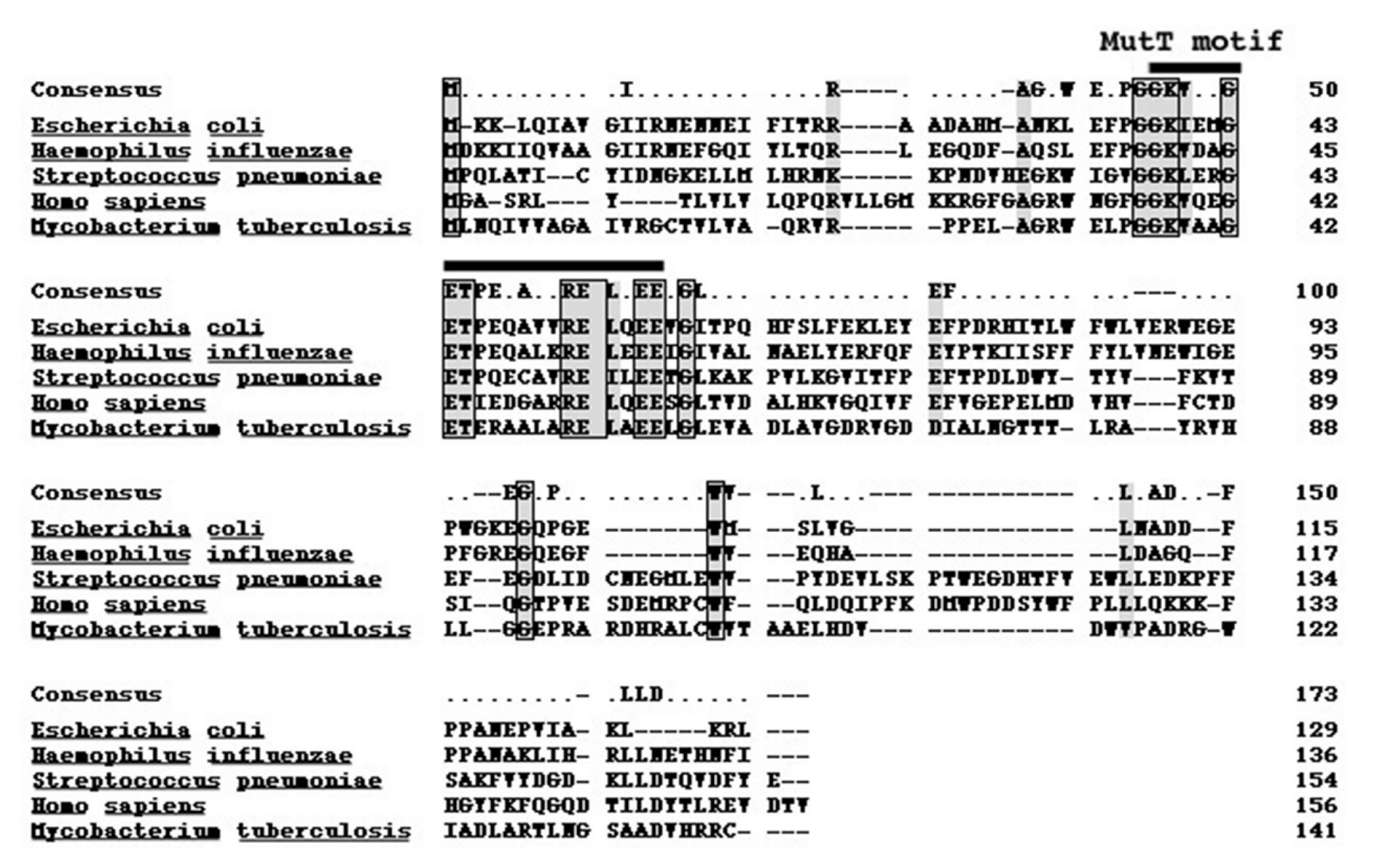
Alignment of the MutT Motif Members of the MutT-related proteins are categorized by a conserved motif originally identified as an important functional motif in the *E. coli* MutT and *S. pneumoniae* MutX antimutator proteins. Similar amino acids are shadowed and identical residues among all species are boxed.

The genomic DNA of *M. tuberculosis* H37Rv was prepared to isolate *mutT* gene. *M. tuberculosis* MutT, encoding a product of 141 amino acid residues with an expected molecular weight of 15.1 kD, was amplified from the genomic DNA of H37Rv with PCR primers designed according to the published sequence (accession no. Z95584, AL 123456 CDS 24794-25219). Sequence of the *mutT* gene was determined on both strands of DNA and was shown to be identical to the reported sequence. The amino acid sequence of MutT protein displays 23% identity to that of *E. coli* MutT with the most sequence similarity in the MutT motif (Figure 1) (16).

**Figure 2.**
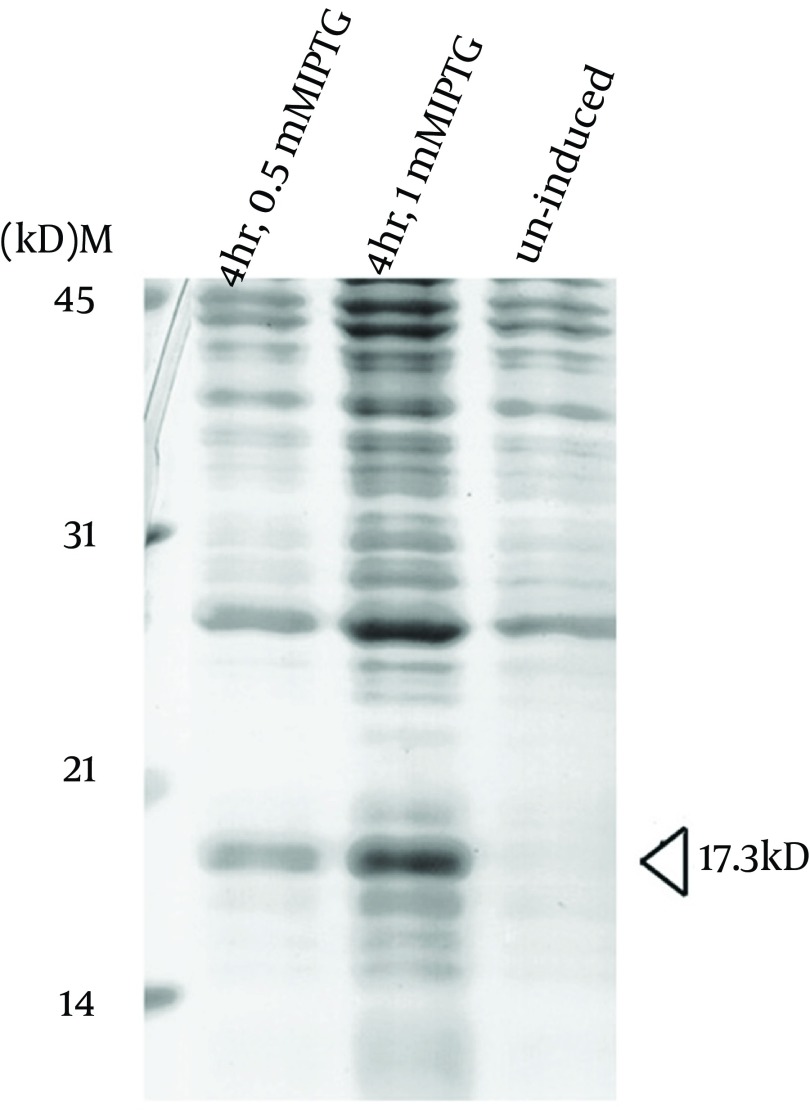
Overexpression of the Recombinant *M. tuberculosis* MutT Protein in BL21 (DE3) Plasmid pET28a (+)-mutT was overexpressed in *E. coli* strain BL21 (DE3) upon IPTG induction to produce the recombinant *M. tuberculosis* MutT protein. Lysates of bacteria were fractionated in a SDS-PAGE gel, and stained with Coomassie Blue. The recombinant *M. tuberculosis* MutT protein of 17.3 kD was found in the fractions.

**Figure 3.**
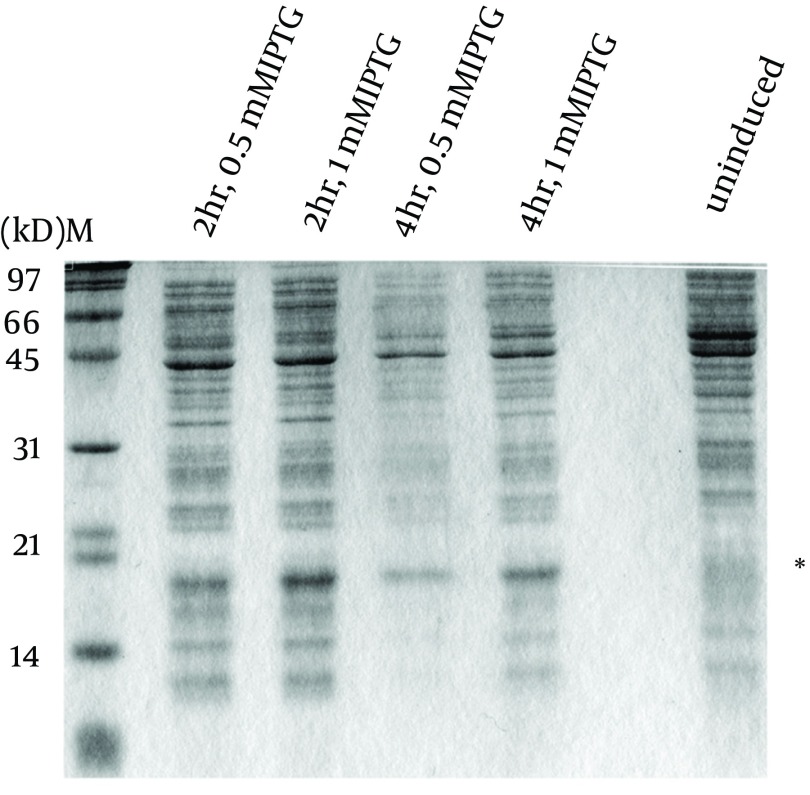
Overexpression of *M. tuberculosis* MutT in MK602 (DE3) *E. coli* strain MK602 (DE3) harboring the plasmid pET28a(+)-mutT was prepared to overexpress the recombinant *M. tuberculosis* MutT protein upon IPTG induction. Lysates of bacteria were subjected to SDS-PAGE and electrotransferred to a nitrocellulose membrane. The membrane was stained by the Ponceau S stain. The recombinant *M. tuberculosis* MutT protein with a molecular weight of 17.3 kD was present; and the asterisk indicates the position of the histidine-tagged *M. tuberculosis* MutT fusion protein.

**Figure 4.**
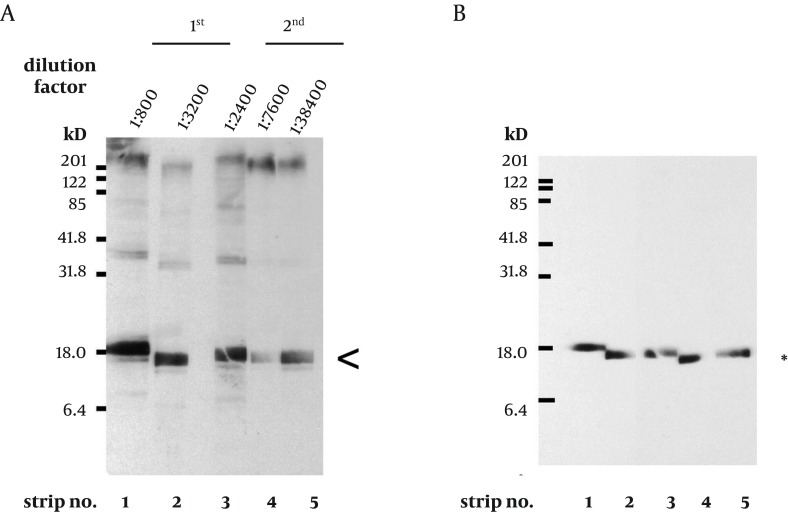
Characterization of the Anti-*M. tuberculosis* MutT Antisera A. Titers of antisera prepared from the same rabbit immunized with purified *M. tuberculosis* MutT are examined using total lysate of *E. coli* strain BL21 (DE3) harboring pET28a (+)-mutT. The batches of antisera were collected on the 6th (1st) and the 7th day (2nd) after the 1st and the 2nd boost immunization, respectively. A prominent signal of the recombinant *M. tuberculosis* MutT protein was still present (arrowhead) even at high dilution factors. B. The same blots were stripped and probed with anti-His antibody (Clontech, 1:5000) to mark the position of the recombinant *M. tuberculosis* MutT protein.

To express the recombinant *M. tuberculosis* MutT protein, the *mutT* gene was subcloned into the expression vector pET28a (+) and the recombinant plasmid was transformed into *E. coli* strains BL21 (DE3) (Figure 2) and MK602 (*mutT*-) (Figure 3). A 17.3-kD fusion protein of MutT with the 6-histidine tag can be expressed upon IPTG induction. The recombinant protein was then purified with an affinity nickel column. The recombinant *M. tuberculosis* MutT protein was used to generate rabbit anti-endogenous *M. tuberculosis* MutT antibody. Titers of antisera were determined with Western blotting using total lysate of *E. coli* strain BL21 (DE3) harboring pET28a (+)-*mutT* (Figure 4). 

**Figure 5.**
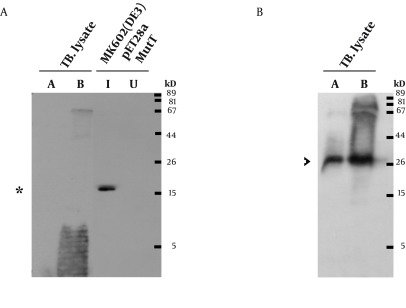
Identification of the Endogenous MutT Protein in the *M. tuberculosis* Lysate A. Lysates of *M. tuberculosis*, MutT induced (I), and uninduced (U) *E. coli* were resolved by SDS-PAGE. They were then analyzed by Western blotting with a 1,40,000 dilution of the rabbit anti-*M. tuberculosis* MutT antiserum. Asterisk indicates the position of the recombinant MutT protein. *M. tuberculosis* lysate A indicates short-term culture and lysate B indicates long-term culture. B. The same membrane was stripped off the antibodies for 25 minutes and reprobed with anti *M. tuberculosis* SOD-C antibody (1:500). Arrowhead indicates the position of the endogenous SOD-C protein.

A prominent signal of the recombinant *M. tuberculosis* MutT protein was still present at high dilution factors of the antibody. This antibody was then used to detect the presence of the endogenous MutT in the *M. tuberculosis* lysate. As shown in Figure 5A, the endogenous MutT protein was observed in *M. tuberculosis* lysate and presented a smear pattern. In addition, a clear band matching the size of the intact endogenous SOD-C protein could be found when the same membrane was reprobed with anti-*M. tuberculosis*-SOD-C antibody (Figure 5B) (17). This indicates that the smear pattern of MutT is not due to gradual protein degradation during lysate preparation.

**Figure 6.**
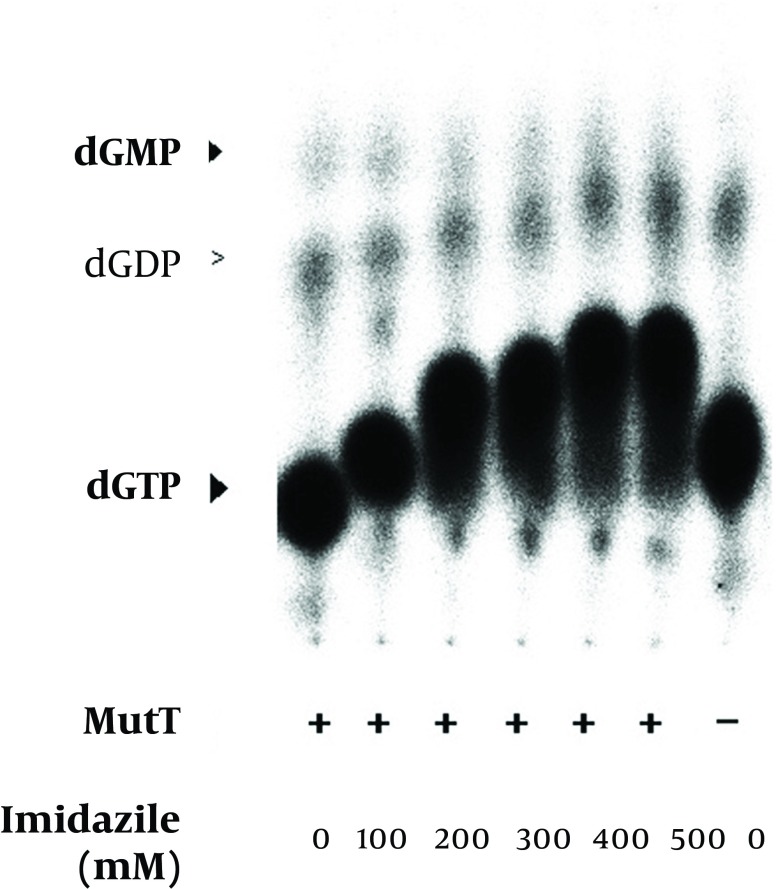
Effect of Imidazole on the Enzymatic Activity of the Recombinant *M. tuberculosis* MutT Protein The renatured MutT was subjected to an increasing concentration of imidazole in the *in vitro* assay. The mobility of nucleotides was changed in the *in vitro* assay with an increasing concentration of imidazole. Positions of dGTP, dGDP, and dGMP were determined by co-migrated unlabeled standards visualized by UV illumination. The negative symbol (-) indicates the reaction without the MutT protein.

**Figure 7.**
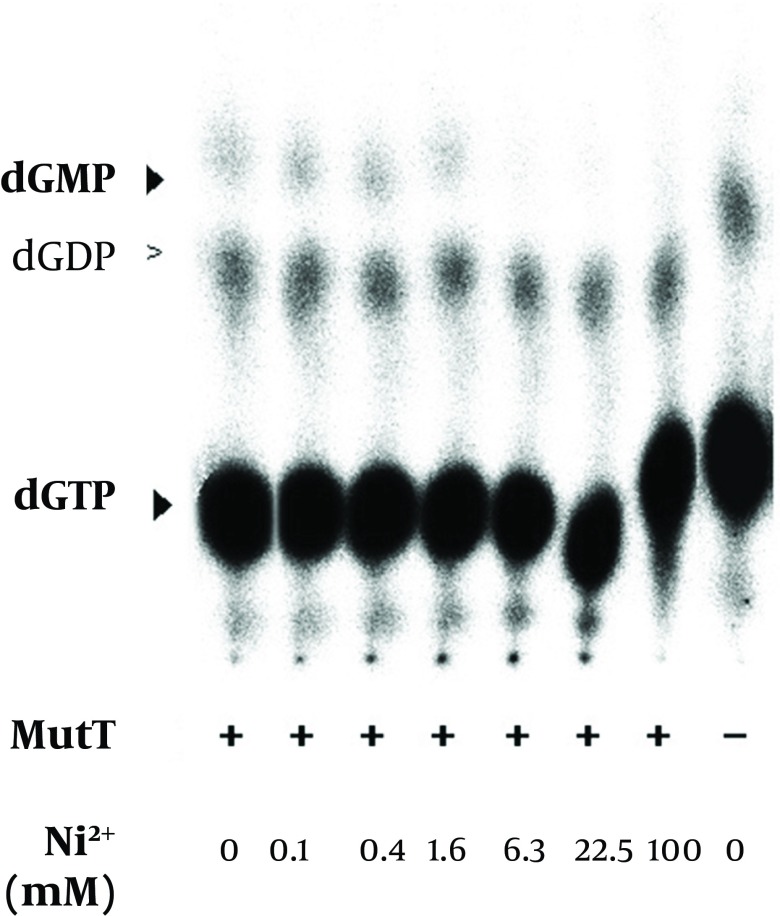
Effect of Nickel on the Enzymatic Activity of Recombinant *M. tuberculosis* MutT Protein The renatured MutT was subjected with an increasing concentration of nickel in the *in vitro* assay. Production of dGMP was reduced with an increasing nickel concentration. The negative symbol (-) indicates the reaction without the MutT protein.

**Figure 8.**
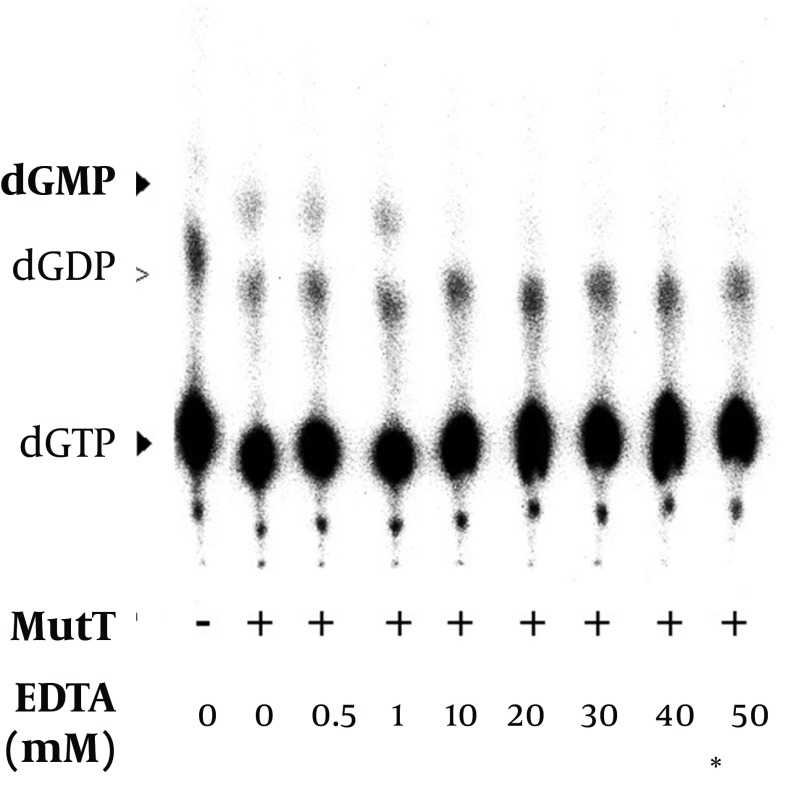
Effect of EDTA on the Enzymatic Activity of the Recombinant *M. tuberculosis* MutT Protein The renatured MutT was subjected to 0-50 mM of EDTA in the *in vitro* assay. The negative symbol (-) indicates the reaction without the MutT protein. Asterisk points out the concentration that we usually use to stop the reaction of the *in vitro* assay.

To demonstrate that the open reading frame of putative *M. tuberculosis* *mutT* gene encodes a functional MutT protein, the MutT (without removing imidazole, Ni^2+^ or other chemicals used in the purification procedure) was applied to an *In vitro* assay (Table 1). Following the procedure (13), we used [α-32P] dGTP as a substrate to assay the putative dGTPase activity of *M. tuberculosis* MutT. The dGTPase activity level was detected (Table 1). The level of dGDP presented as a background value, i.e. there was hydrolysis of dGTP to dGDP even when there was no *M. tuberculosis* MutT protein in the reactions (Figures 6, 7 and 8).

**Table 1.** The dGTPase Activity of *M. tuberculosis* MutT Protein Before and After the Removal of Purification Reagents ^a^

dGTP	dGMP		dGDP	
PSL	PSL	Percent, Mean ± SE	PSL	Percent, Mean ± SE
**Purification of *M. tuberculosis* MutT with Ni^2+^**				
35198.01	285.30	0.79 ± 0.02 ^b^	1433.30	4.03 ± 0.16
35651.44	277.09		1527.85	
35837.54	279.94		1338.91	
**Refolding of ** ***M. tuberculosis*** ** MutT**				
35439.42	581.65	1.46 ± 0.15 ^b^	1435.31	3.78 ± 0.14
35656.86	495.68		1303.84	
35678.71	484.80		1292.87	

^a^ Abbreviations: dGDP, deoxyguanosine diphosphate; dGMP, 2'-deoxyguanosine 5'-phosphate; dGTP, deoxyguanosine triphosphate; PSL, photo-stimulated luminescence.

^b^ Percentages differ significantly (P < 0.05)

This result may reflect that the purification reagents like nickel, can reduce the activity of *M. tuberculosis* MutT. Subsequently, denaturation and renaturation procedures were used to remove contaminating reagents and proteins. The *M. tuberculosis* MutT was fractionated in a SDS-PAGE gel and visualized by KCl staining. The gel containing the recombinant *M. tuberculosis* MutT protein was cut out and ground into small pieces. The protein was eluted from the gel pieces, and renatured to form *M. tuberculosis* MutT protein of high purity. As shown in Table 1, the dGTPase activity of recombinant *M. tuberculosis* MutT significantly increased (P < 0.05) after renaturation. The level of dGDP was still present as a background value and did not show significant difference after refolding. 

The refolded MutT was then used to test the effects of different reagents in the purification procedure. The histidine-tagged MutT protein was eluted from the Ni^2+^-charged column with 260-400 mM imidazole linear gradient. To examine the imidazole effect on the enzymatic activity of *M. tuberculosis* MutT protein, fractions isolated from increasing imidazole concentration of 0-400 mM were added to the *In vitro* activity assay. The mobility of nucleotides changed (Figure 6) but imidazole presence did not significantly affect the dGTPase activity of *M. tuberculosis* MutT protein. The refolded MutT protein was also applied to estimate the dGTPase activity with nickel ion addition. The dGMP production was attenuated by nickel addition (Figure 7). Even at concentrations as low as 6.3 mM (the concentration that the manufactureer’s instructions recommend to charge the affinity column is 100 mM), we observed reduction of dGMP production. Therefore, this showed that the dGTPase activity of the Ni^2+^-charged-column purified *M. tuberculosis* MutT may be partially inhibited by the existence of nickel. 

**Figure 9.**
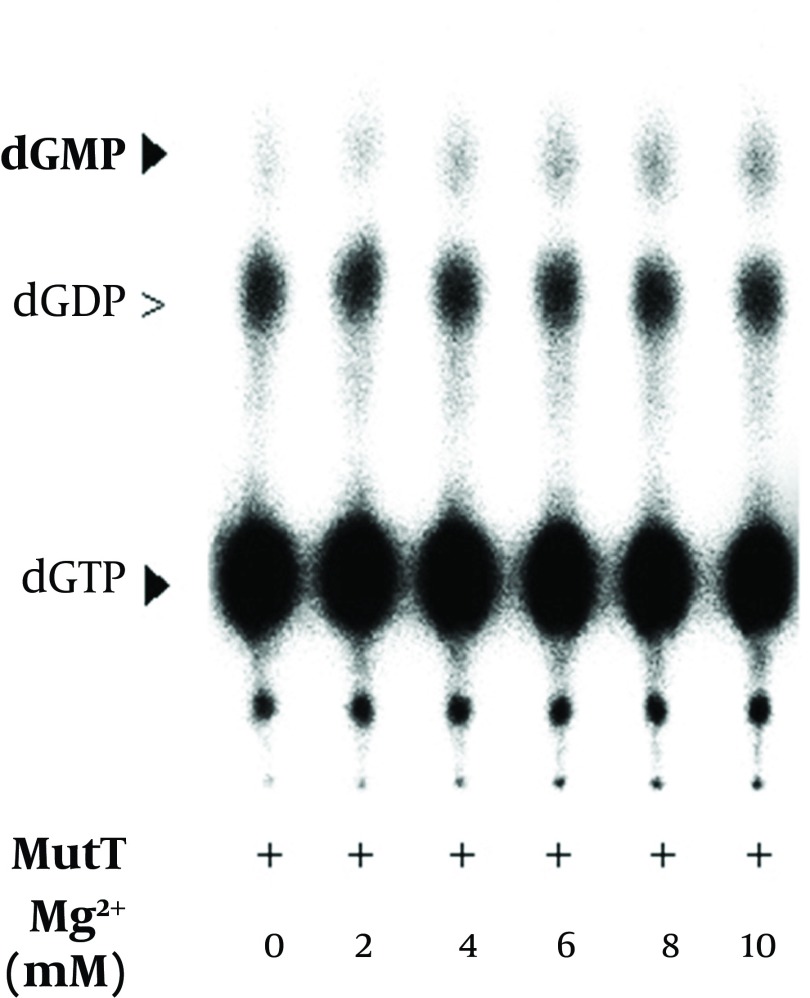
Effect of Magnesium on the Enzymatic Activity of the Recombinant *M. tuberculosis* MutT Protein The MutT *in vitro* assays were performed with an increasing concentration of magnesium in the *in vitro* assay.

It has been previously reported that magnesium is a cofactor of *E. coli* MutT in its dGTPase activity (18). In the *In vitro* assay, we found that dGMP production increased with the concentration of magnesium (Figure 9). The level of dGDP presented as a background value and did not show significant difference in the presence of magnesium. The results indicated that the *M. tuberculosis* MutT activity was enhanced by an increased magnesium concentration. In our dGTPase activity assay, a final EDTA concentration of 45 mM was generally used to stop the reaction. Figure 8 shows that a concentration as low as 10 mM of EDTA can inhibit hydrolysis of dGMP as low as the negative control level. Therefore, treatment of EDTA could stop the reaction almost completely in the dGTPase activity assay.

## 5. Discussion

The MutT protein can prevent DNA transversion by hydrolysis of 8-oxo-dGTP. It can also hydrolyze all canonical nucleoside triphosphates or deoxynucleoside triphosphates. When the regulation of MutT is lost, it may accumulate excess MutT protein to remove normal nucleotides instead of prevention of DNA abnormal changes. For some important transcription factors, rapid degradation is a pathway to regulate their function. One of the well-known cases is p53 (19): it has a very short half-life and is tightly regulated by protein degradation (20). In the assay of probing endogenous MutT protein, we observed that the endogenous MutT protein displays a smear pattern. In addition, the presence of intact endogenous SOD-C protein, as shown by probing the same membrane, rules out the possibility of spontaneous degradation. Thus we speculated that the endogenous MutT protein was unstable and regulated by degradation. An excellent substrate of *E. coli* MutT protein is dGTP. In fact, the *E. coli* MutT protein has the highest Vmax on dGTP when compared to other deoxyribonucleotides, ribonucleotides or oxidative-damaged nucleotides (4). In the *In vitro* assay, we observed that the dGTP loss comes with the formation of dGMP, similar to the results found for *E. coli* described by Bhatnagar et al. (3).

The MutT protein is a repair enzyme with dGTPase activity (2). It has been speculated that organisms living under severe oxidative stress, like *M. tuberculosis*, should have effective systems to repair oxidative DNA damages. Here we cloned and characterized *M. tuberculosis* *mutT* in an attempt to elucidate the importance of MutT in *M. tuberculosis*. We found that *M. tuberculosis* MutT had a dGTPase activity, suggesting that *M. tuberculosis* MutT is a functional homologue of *E. coli* MutT. Because the repair enzyme feature of the MutT protein should be critical for survival of M. tuberculosis, it is conceivable that the DNA repair system *M. tuberculosis* could relate to a pathogenic process. If function loss by mutagenesis or knockout of *mutT* gene can abolish or reduce the virulence of *M. tuberculosis*, it will provide evidence of the relationship between the virulence of the pathogen and the DNA repair system.
